# Supplementary material for: MARCH family E3 ubiquitin ligases selectively target and degrade cadherin family proteins
Source: bioRxiv. 2023 Aug 10:2023.08.10.552739. Preprint. [Version 1] doi: 10.1101/2023.08.10.552739 (PMC10441400; doi:10.1101/2023.08.10.552739)
Supplement: 1 [file NIHPP2023.08.10.552739V1-supplement-1.pdf]

bioRxiv preprint doi: https://doi.org/10.1101/2023.08.10.552739; this version posted August 10, 2023. The copyright holder for this preprint (which was not certified by peer review) is the author/funder, who has granted bioRxiv a license to display the preprint in perpetuity. It is made available under aCC-BY 4.0 International license.

Supporting information

Fig S1. E-cadherin localization in MARCH-transfected cells. A431 cells were transfected with MARCH family proteins and subjected to immunofluorescence with E-cad antibody then processed for fluorescence microscopy. Transfected cells are indicated with yellow arrowhead. Scale bar = 25 μm

Fig S2. Cadherin chimeras localize to cell-cell junctions in A431 cells.

bioRxiv preprint doi: https://doi.org/10.1101/2023.08.10.552739; this version posted August 10, 2023. The copyright holder for this preprint (which was not certified by peer review) is the author/funder, who has granted bioRxiv a license to display the preprint in perpetuity. It is made available under aCC-BY 4.0 International license.

Localization of RFP tagged various cadherin chimeras were assessed in A431 cells expressing GFP. Scale bar = 50 µm

Fig S3. Localization of cell adhesion molecules in A431 cells and A431 E-cadherin/P-cadherin null cells. Wild type A431 cells or A431 cells lacking E- and P-cadherin [43] were processed for fluorescence microscopy to localize E-cadherin, N-cadherin, β-catenin or p120-catenin. The lack of β-catenin and p120-catenin at cell-cell contacts confirms lack of other classical cadherins. Scale bar = 50 µm

Fig S4. Localization of VE-cadherin mutants lacking membrane proximal lysine residues. E-cadherin/P-cadherin null A431 cells expressing various VE-cadherin mutants were analyzed by immunofluorescence for localization of the cadherin and p120-catenin in cells expressing GFP. Scale bar = 25 µm
